# Self-Assembled Protein–Polymer Nanoparticles via Photoinitiated Polymerization-Induced Self-Assembly for Targeted and Enhanced Drug Delivery in Cancer Therapy

**DOI:** 10.3390/molecules30040856

**Published:** 2025-02-13

**Authors:** Gayathri R. Ediriweera, Yixin Chang, Wenting Yang, Andrew K. Whittaker, Changkui Fu

**Affiliations:** 1 Australian Institute for Bioengineering and Nanotechnology, The University of Queensland, St. Lucia, QLD 4072, Australia; a.ediriweera@uq.edu.au (G.R.E.); yixin.chang@uq.edu.au (Y.C.); wenting.yang2@student.uq.edu.au (W.Y.); 2 Australian Research Council Centre of Excellence for Green Electrochemical Transformation of Carbon Dioxide, The University of Queensland, St. Lucia, QLD 4072, Australia

**Keywords:** protein-polymer nanoparticles, photo-PISA, drug delivery, cancer therapy

## Abstract

Protein–polymer bioconjugates offer numerous advantages in biomedical applications by integrating the benefits of functional proteins and tunable synthetic polymers. Developing drug-loaded protein–polymer nanoparticles, with a receptor-targeting protein forming the nanoparticle shell, would be ideal for the targeted delivery of drugs to cancer cells that overexpress specific receptors for more effective cancer therapy. In this study, we report the synthesis of reduction-responsive protein–polymer nanoparticles by a photoinitiated polymerization-induced self-assembly (photo-PISA) approach. Anti-cancer drugs can be efficiently encapsulated at high concentrations within the nanoparticles during the photo-PISA process. These protein–polymer nanoparticles present transferrin (Tf) on their surfaces, capable of targeting the overexpressed Tf receptors found on cancer cells. It was found that the nanoparticles demonstrate enhanced cellular uptake and delivery of the anti-cancer drug, curcumin, to cancer cells via Tf receptor-mediated endocytosis, compared to the control PEGylated nanoparticles that lack targeting capability. Moreover, the nanoparticles can release the encapsulated curcumin in response to a reducing environment, a characteristic of cancer cells compared to health cells. Consequently, the synthesized protein–polymer nanoparticles are more effective in inducing cancer cell death compared to the control nanoparticles, demonstrating their potential as an effective and targeted drug delivery system for cancer therapy.

## 1. Introduction

Biomolecule–polymer conjugates are an important class of hybrid materials that combine the favourable properties of natural biomolecules with those of synthetic polymers [1,2,3]. They have been employed in a variety of biomedical, antimicrobial, and catalytic applications [4,5,6]. Among various biomolecules employed in these applications, proteins are recognized as a promising class of pharmaceuticals owing to their precise binding interactions and limited off-target effects [7]. Hence, protein–polymer conjugates have emerged as an important class of biohybrid material for biomedical applications [8,9,10]. Using controlled polymerization methods along with “grafting-to”, “grafting-from”, or “grafting-through” approaches is the key to synthesizing numerous biomolecule–polymer hybrids to serve the intended purposes [11]. One such bioconjugate synthesis strategy involves the conjugation of hydrophobic polymers onto hydrophilic proteins to generate amphiphilic bioconjugates that can self-assemble in aqueous solutions to form highly ordered protein nanoparticles [7,11]. The resulting bioconjugate can contain a high density of proteins on the nanoparticle surface, acting as the hydrophilic shell and providing the opportunity to maximize the beneficial traits of the biomolecule, such as achieving enhanced targeting efficacy. Moreover, the properties of the nanoparticle, including size and morphology, can be readily engineered by modifying the length of the hydrophobic polymer attached to the protein [1,7].

The traditional method of the preparation of amphiphilic protein–polymer conjugates involve the covalent conjugation of a hydrophobic polymer to a hydrophilic protein, typically in an organic solvent via a “grafting-to” method and subsequent self-assembly of the amphiphile in aqueous solution [7,12,13]. Due to the use of organic solvents, this method often results in denaturation or loss of protein activity and is further complicated by challenges in controlling the self-assembly process and ensuring proper purification [14]. Hence, there is a significant interest in the development of new synthetic strategies that can circumvent these limitations. Recently, polymerization-induced self-assembly (PISA) has received significant attention in the development of biomolecule–polymer nanoparticles [7,15,16]. More specifically, atom-transfer radical PISA (ATR-PISA) has been utilized by Gao et al. and others to develop biomolecule–polymer hybrid nanoparticles, mostly involving a protein as the biomolecule for therapeutic protein delivery applications [17,18,19]. Compared to ATR-PISA, reversible addition–fragmentation chain transfer (RAFT)-PISA is considerably less explored for the preparation of protein–polymer nanoparticles. The use of the traditional thermal RAFT method is usually not suitable for protein conjugation since it could lead to protein denaturation [16]. The recent development of photo-initiated RAFT (photo-RAFT) polymerization could be ideal for the synthesis of bioconjugates since the polymerization can be performed under mild conditions involving aqueous buffers and at room temperature to keep the protein intact [20,21,22,23]. However, the use of photo-RAFT-mediated PISA to develop self-assembled protein nanoparticles for biomedical applications is surprisingly less explored.

**Figure 1:**
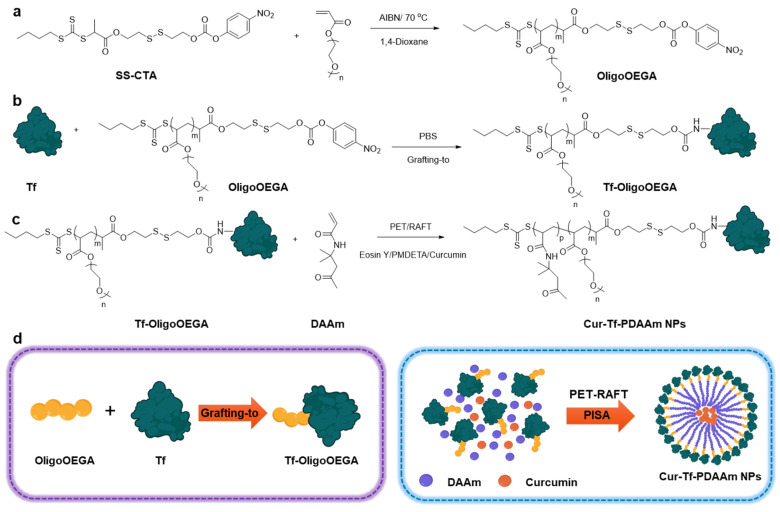
Reaction scheme for the synthesis of (**a**) an oligomer containing reduction-responsive CTA (OligoOEGA); (**b**) transferrin-oligomer macro-CTA (Tf-OligoOEGA); and (**c**) curcumin-loaded transferrin–polymer nanoparticles (Cur-Tf-PDAAm nanoparticles); (**d**) graphical representation of the synthesis of Tf-OligoOEGA and Cur-Tf-PDAAm nanoparticles (created with Biorender.com).

In this study, we report the synthesis of reduction-responsive, anti-cancer drug-loaded protein–polymer nanoparticles using photoinduced electron/energy transfer reversible addition–fragmentation chain transfer polymerization [24] (PET-RAFT)-mediated PISA (photo-PISA) for targeted cancer therapy (Figure 1). Transferrin (Tf) protein was employed as the hydrophilic protein component in the nanoparticles since it can target the Tf receptors that are overexpressed in a variety of cancer cells [25]. A macro-chain transfer agent (CTA) was first synthesized by conjugating a Tf protein to a poly(ethylene glycol) methyl ether acrylate (OEGA) oligomer (OligoOEGA) containing a reduction-sensitive disulfide linker via a “grafting-to” method. Since the tumour site is associated with a reducing environment due to an elevated glutathione (GSH) concentration compared with normal cells, the incorporation of a disulfide linker to the nanocarriers can allow for their reduction-responsive degradation in tumour cells and the release of loaded cargos [26]. Subsequent PET-RAFT polymerization with Tf-OligoOEGA macro-CTA and diacetone acrylamide (DAAm) core-forming monomer in an aqueous buffer resulted in a PISA process and the generation of spherical nanoparticles with Tf constituting the shell. Hydrophobic anti-cancer drugs, such as curcumin, can also be successfully encapsulated in the nanoparticles during the photo-PISA process. The Tf-based nanoparticles can respond to elevated levels of GSH by cleaving the disulfide linkages, thereby enhancing drug release within cancer cells. By effectively targeting the Tf receptors overexpressed by breast cancer cells, the nanoparticles exhibited improved cancer cell uptake and chemotherapy effects compared to non-targeted nanoparticles. Therefore, the protein–polymer nanoparticles generated through PET-RAFT-mediated photo-PISA demonstrate great potential to serve as a targeted drug delivery system for a more effective cancer therapy.

## 2. Results and Discussion

We used a combination of “grafting to” and “grafting from” methods to synthesize a Tf-based macro-CTA for mediating PISA (Figure 1). A small molecule CTA containing a reduction-responsive disulfide group and an amine-reactive 4-nitrophenyl carbonate group was first prepared (SS-CTA, Figure S1). The SS-CTA was thoroughly characterized using ^1^H and ^13^C NMR as well as mass spectrometry (Figures S2–S5). The reduction sensitivity of SS-CTA was then investigated by incubating in a 10 mM GSH solution to mimic the reducing intracellular tumour microenvironment [26]. Cleavage of the disulfide bond was evaluated by ^1^H NMR by comparing the integrals of peaks in the aromatic regions for the initial SS-CTA (8.3 and 7.5 ppm) and the degraded products (8.1 and 6.9 ppm) (Figure S6). Within 24 h, >80% of disulfide bond cleavage was achieved according to the ^1^H NMR analysis. This reduction-sensitive disulfide bond cleavage of SS-CTA was further confirmed using LC-MS analysis (Figure S7).

SS-CTA is hydrophobic and cannot be used for conjugation with Tf in an aqueous solution. An oligomer of poly(ethylene glycol) methyl ether acrylate (OligoOEGA) was then synthesized by thermal RAFT polymerization using SS-CTA to obtain a water-soluble macro-CTA for subsequent protein conjugation (Figure 1a). The OligoOEGA was characterized by ^1^H NMR, which revealed an average degree of polymerization (DP) of ~6 and a molecular weight of 3460 g/mol (Figure S8). The OligoOEGA showed a narrow molecular weight distribution with a dispersity of 1.15 according to size exclusion chromatography (SEC) analysis (Figure S9). The OligoOEGA was subsequently conjugated to Tf in phosphate-buffered saline (PBS) to yield Tf-based macro-CTA (Tf-OligoOEGA) (Figure 1b). The successful synthesis of Tf-OligoOEGA was confirmed by ^1^H NMR showing the presence of characteristic peaks of OligoOEGA in the protein conjugate (Figure S10) and the increase in hydrodynamic size of the Tf from ~4 nm to ~7 nm measured by dynamic light scattering (DLS) (Figure S11). Moreover, MALDI-TOF analysis confirmed the presence of ~2.4 OEGA chains per protein (Figure S12). Furthermore, the successful conjugation of OligoOEGA to Tf was verified by SDS-PAGE, as indicated by the appearance of a new tailing band that corresponds to Tf-OligoOEGA with a higher molecular weight (Figure S13).

**Figure 2:**
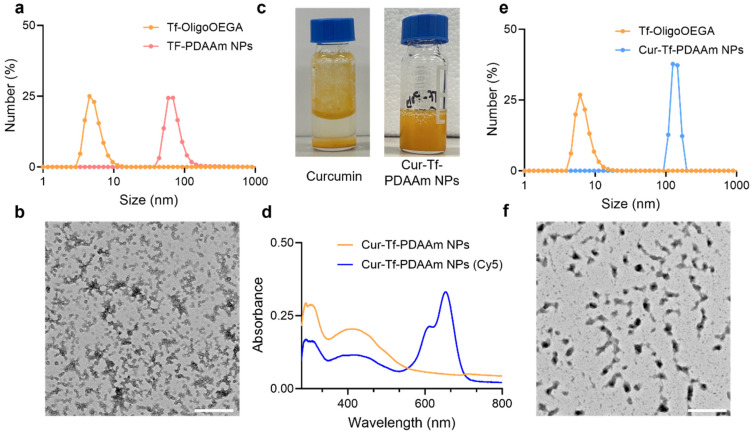
(**a**) Hydrodynamic size and (**b**) TEM image of Tf-PDAAm nanoparticles prepared by photo-PISA (scale bar: 500 nm); (**c**) photographs of free curcumin and Cur-Tf-PDAAm nanoparticles in PBS buffer; (**d**) UV-Vis spectra of Cur-Tf-PDAAm and Cur-Tf-PDAAm (Cy5) nanoparticles in PBS; (**e**) hydrodynamic size and (**f**) TEM image of Cur-Tf-PDAAm nanoparticles (scale bar: 500 nm).

Using Tf-OligoOEGA as the hydrophilic macro-CTA and diacetone acrylamide (DAAm) as the core-forming monomer [27], an aqueous photo-PISA was conducted under the irradiation of blue light (solid content: 8%, [Tf-OligoOEGA]:[DAAM]:[Eosin Y]:[N,N,N′,N″,N″-Pentamethyldiethylenetriamine (PMDETA)] = 1:250:0.25:1, λ_max_ = 470 nm, Figure 1c). After polymerization for 5 h, a monomer conversion of ~80% was achieved and the solution changed from a transparent solution to a turbid suspension, a typical phenomenon in PISA indicative of successful formation of nanoparticles. After purification by dialysis, the obtained nanoparticles were characterized by ^1^H NMR (Figure S14), DLS (Figure 2a), and transmission electron microscopy (TEM) (Figure 2b). The NMR spectrum confirmed the successful chain extension with DAAm by PET-RAFT polymerization. The DLS measurement showed that the nanoparticles had a hydrodynamic radius of ~70 nm compared with ~7 nm of Tf-OligoOEGA. TEM revealed that the nanoparticles presented a uniform spherical vesicle morphology. In summary, the results confirmed the successful formation of Tf-based nanoparticles through a photo-PISA process.

After demonstrating that Tf-based nanoparticles can be prepared via a photo-PISA approach, a similar method was used to prepare anti-cancer drug-loaded Tf-based nanoparticles. In this work, we chose to use curcumin as a model drug for in situ encapsulation via the photo-PISA process. Curcumin is a biologically active compound with numerous pharmacological benefits, including anti-cancer, anti-inflammatory, and anti-oxidant activity [28]. However, the benefits of curcumin are hindered by its poor water solubility, rapid liver metabolism as well as limited systemic circulation [28,29]. Formulation of curcumin into nanoparticles can potentially overcome these challenges and improve the systemic delivery of curcumin for cancer therapeutic applications. Curcumin was added to the photo-PISA system of Tf-OligoOEGA and DAAm to prepare the curcumin-loaded Tf-PDAAm nanoparticles (Cur-Tf-PDAAm NPs). While free curcumin is not soluble in PBS due to its highly hydrophobic nature (Figure 2c), it forms a clear and homogeneous suspension after the photo-PISA reaction, indicating the successful encapsulation of curcumin within the formed nanoparticles. This was further confirmed by UV-vis spectroscopy showing the characteristic absorbance peak of curcumin at 425 nm (Figure 2d). The drug loading efficiency was determined to be ~30%. The hydrodynamic size of the drug-loaded Cur-Tf-PDAAm NPs was found to be larger (~125 nm) than that of Tf-PDAAm NPs without drug encapsulation (~70 nm) (Figure 2e). The encapsulation of curcumin resulted in a change in the morphology of the nanoparticles. The Cur-Tf-PDAAm nanoparticles exhibited micellar structures with a relatively irregular spherical shape (Figure 2f), in contrast to the uniform round vesicles formed in the absence of curcumin (Figure 2b). This implies that the hydrophobic curcumin interfered with the PISA process and, hence, influenced the resultant morphologies, as previously reported [30]. The Cur-Tf-PDAAm NPs were fluorescently labelled by reacting a proportion of the amine groups on the protein with Cy5-NHS ester to facilitate visualization by confocal microscopy in subsequent in vitro investigations (Figure 2d).

For comparison with the reduction-responsive Cur-Tf-PDAAm nanoparticles, polymeric nanoparticles without active targeting (i.e., without Tf protein) and reduction-responsive drug release capability were also prepared. Accordingly, a POEGA-based macro-CTA with a similar molecular weight to Tf-OligoOEGA was first synthesized using the SS-CTA (Figure S15). An azide-containing monomer was also incorporated into the polymer chain during the RAFT polymerization for subsequent dye labelling. The number average molecular weight of the POEGA macro-CTA was determined to be 96,060 g/mol according to ^1^H NMR analysis (Figure S16), which was slightly higher than the molecular weight of Tf-OligoOEGA macro-CTA (84,370 g/mol). The POEGA macro-CTA exhibited a narrow molecular weight distribution with a dispersity of 1.11 determined by SEC. Afterward, the POEGA macro-CTA was used for PET-RAFT polymerization of DAAm in the presence of curcumin to develop the control curcumin-loaded POEGA-PDAAm nanoparticles (Cur-POEGA-PDAAm NPs). The PEGylated Cur-POEGA-PDAAm NPs were around 120 nm in size according to DLS (Figure S17) and the drug loading efficiency was found to be ~60%. The POEGA-PDAAm NPs were then fluorescently labelled by reacting the azide-containing monomer in the hydrophilic shell with sulfo-Cy5-DBCO (Figure S18).

**Figure 3:**
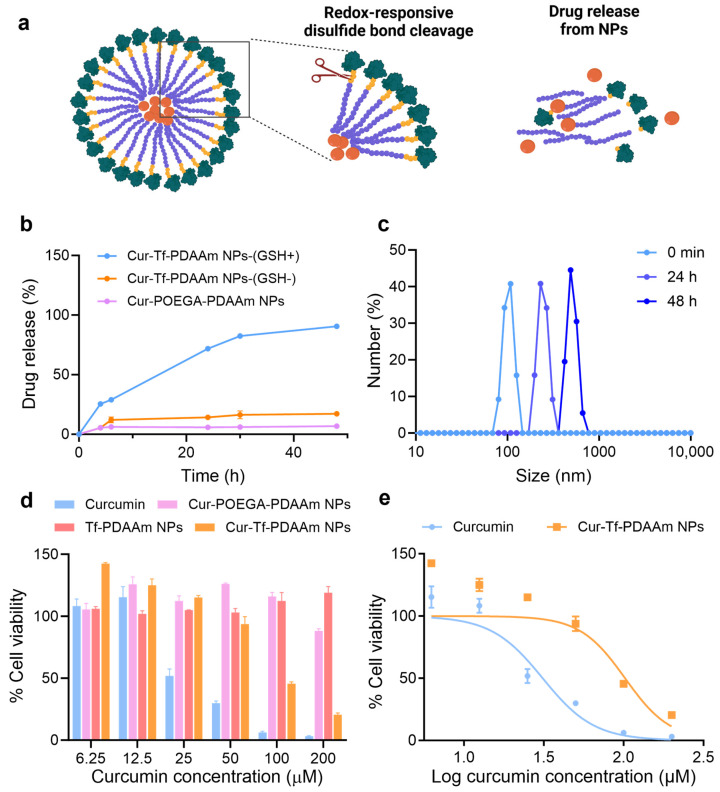
(**a**) A diagram demonstrating the reduction-responsive drug release from Cur-Tf-PDAAm nanoparticles (created with Biorender.com); (**b**) cumulative drug release from Cur-Tf-PDAAm and Cur-POEGA-PDAAm nanoparticles at 37 °C under different conditions; (**c**) DLS analysis of Cur-Tf-PDAAm nanoparticles incubated with 10 mM GSH; (**d**) cell viability of MDA-MB-231 cells treated with Cur-Tf-PDAAm nanoparticles, Cur-POEGA-PDAAm nanoparticles, Tf-PDAAm nanoparticles and free curcumin determined by the MTS assay; (**e**) IC_50_ determination of Cur-Tf-PDAAm NPs and curcumin using MDA-MB-231 cells.

The reduction-responsive drug release of Cur-Tf-PDAAm NPs was then investigated by incubating the particles in a 10 mM GSH solution mimicking the high reducing environment in cancer cells (Figure 3a) [31]. A cumulative release of ~90% curcumin from the Cur-Tf-PDAAm NPs was observed within 48 h when incubated in 10 mM GSH containing a buffer in contrast to the control Cur-POEGA-PDAAm NPs from which less than 10% drug release was observed (Figure 3b). In addition, the Cur-Tf-PDAAm NPs only released 17% of the encapsulated curcumin in the absence of GSH (Figure 3b). This demonstrated that the reduction-responsive drug release behaviour of Cur-Tf-PDAAm NPs is attributed to the GSH-induced cleavage of the disulfide bonds connecting the hydrophilic protein and the hydrophobic PDAAm segments, thereby leading to the disassembly of the NPs and the release of encapsulated curcumin. This was further confirmed by an increase in particle size observed by DLS for Cur-Tf-PDAAm NPs when incubated in the 10 mM GSH solution (Figure 3c). The increase in size is ascribed to the aggregation of the generated hydrophobic polymers after GSH-induced disassembly of the Cur-Tf-PDAAm NPs in the aqueous solution [32,33]. In contrast, the control Cur-POEGA-PDAAm NPs remained stable without any apparent change in size (Figure S19). Furthermore, the Cur-Tf-PDAAm NPs were stable in PBS and serum containing the buffer for a period of 48 h in the absence of GSH (Figure S20), demonstrating their ability to maintain good structural stability, thereby potentially minimizing unwanted drug leakage into healthy cells and enabling targeted drug release in tumour tissue with a reducing environment [31,34,35]. This will help reduce the side toxic effects of the drug delivery system to healthy tissues, a crucial requirement for developing cancer treatment platforms with greater efficacy [36].

The in vitro cytotoxicity of the NPs in comparison to free curcumin was evaluated using an MTS assay on MDA-MB-231 cells. The MDA-MB-231 breast cancer cell line is one of the most-studied cancer cell lines reported to overexpress the Tf receptor on the cell surface [37]; hence, it was adopted in the current in vitro study. The empty vehicle (Tf-PDAAm NPs) did not show cytotoxicity to the MDA-MB-231 cells or normal cells, such as RAW264.7 cells (Figure S21a), confirming the safety of the nanocarrier, which is essential for drug delivery applications [38]. The Cur-Tf-PDAAm treated cells displayed a significant reduction in viability after 48 h incubation compared to the control Cur-POEGA-PDAAm NPs (Figure 3d). This could be attributed to the potentially higher uptake of Cur-Tf-PDAAm NPs into the cancer cells through Tf-receptor mediated endocytosis, along with the successful reduction-responsive intracellular release of curcumin, in contrast to the control Cur-POEGA-PDAAm NPs. Curcumin is reported to cause cancer cell death through the modulation of numerous cell signalling pathways leading to cell apoptosis [39]. The significantly lower cancer cell death caused by Cur-POEGA-PDAAm NPs could be due to their lower uptake into the cancer cells compared to Cur-Tf-PDAAm NPs and a lack of a reduction-responsive drug release mechanism, as observed in Figure 3b. Furthermore, compared to free curcumin, which demonstrated an IC_50_ value of 31 µM, the Cur-Tf-PDAAm NPs exhibited a higher IC_50_ value of up to 102 µM (Figure 3e). This difference could be due to the availability and pharmacodynamics of curcumin: free curcumin is readily accessible to cells, while the Cur-Tf-PDAAm NPs must first be internalized, releasing curcumin over time for eventual metabolism [40]. Additionally, the IC_50_ of the Cur-Tf-PDAAm NPs was also tested on RAW264.7 cells, showing a higher value of 162.1 µM compared to that on MDA-MB-231 cancer cells (Figure S21b). This lower toxicity to normal cells is expected, as RAW264.7 cells lack the reductive intracellular environment found in cancer cells to facilitate the enhanced release of curcumin, and they do not have overexpressed Tf receptors on their surface, which would otherwise increase the uptake of the nanoparticles.

**Figure 4:**
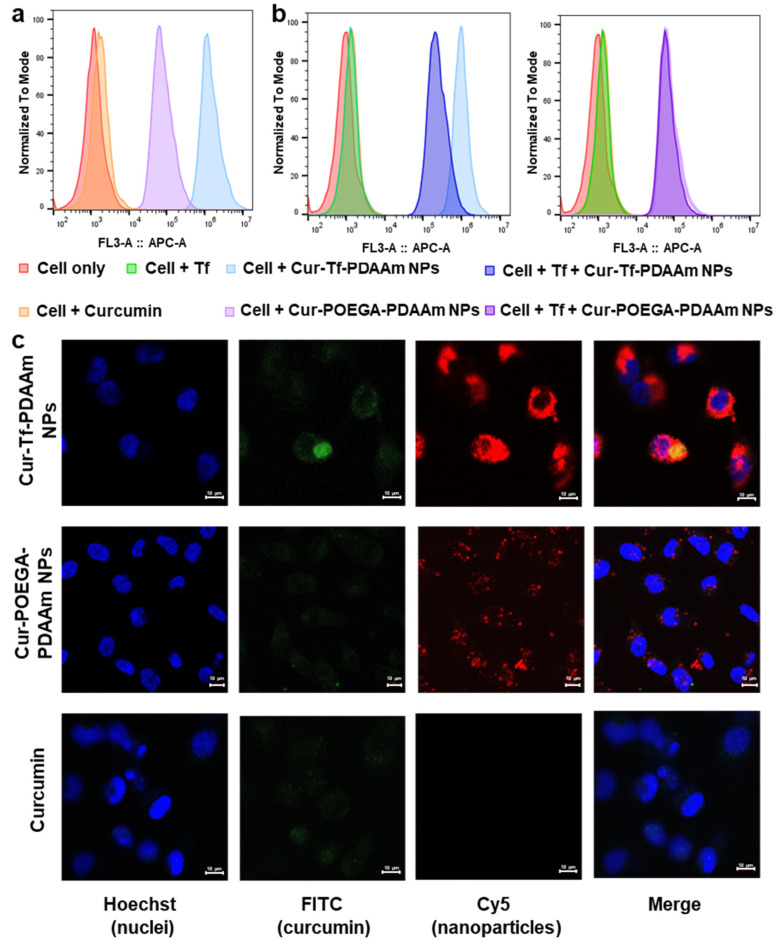
(**a**) Cellular association of Cur-Tf-PDAAm nanoparticles and Cur-POEGA-PDAAm nanoparticles with MDA-MB-231 cells determined by flow cytometry (Cy5 channel); (**b**) cellular association of Cur-Tf-PDAAm nanoparticles and Cur-POEGA-PDAAm nanoparticles with MDA-MB-231 cells determined by flow cytometry (Cy5 channel) after blocking the Tf receptors on cells with excess Tf protein; (**c**) confocal micrographs demonstrating the cellular internalization of Cur-Tf-PDAAm nanoparticles, Cur-POEGA-PDAAm nanoparticles and free curcumin after 5 h incubation with MDA-MB-231 breast cancer cells. Scale bar: 10 µm.

In order to verify the cell viability results that correspond to the uptake of NPs into cancer cells, a cellular association study was conducted using flow cytometry by incubating the MDA-MB-231 cells with Cur-Tf-PDAAm and Cur-POEGA-PDAAm NPs for 1 h. As evident from Figure 4a, higher cellular association was observed with Cur-Tf-PDAAm NPs compared to the control NPs, indicating that the higher effectiveness of Cur-Tf-PDAAm NPs to promote cancer cell death is likely a result of higher cellular uptake of those compared to the control NPs. To further confirm that the higher association of Cur-Tf-PDAAm NPs with cancer cells is due to the Tf-receptor targeting, a blocking experiment was performed. A competitive inhibition study was conducted by incubating the MDA-MB-231 cells with drug-loaded NPs in the presence and absence of excess free Tf protein that can effectively block the Tf receptors on the cancer cell surface. There was an obvious shift in the flow cytometry histogram towards low cellular association in the case of Cur-Tf-PDAAm NPs when incubated with excess Tf protein, confirming that the Tf-receptor targeted by Cur-Tf-PDAAm NPs contributes to the enhanced cellular association (Figure 4b). Conversely, the histograms of Cur-POEGA-PDAAm NPs were comparable in the presence and absence of the free Tf protein, indicating the absence of any cellular association of the NPs based on active targeting (Figure 4b), consequently confirming the effectiveness of utilizing targeted nanocarriers for improving the drug delivery efficiency to the cancer site.

The cellular uptake and internalization of NPs were subsequently investigated using confocal microscopy. In agreement with the flow cytometry results, the Cur-Tf-PDAAm NPs showed a significantly higher cellular internalization into the cytoplasm after 1 h and 5 h of incubation with obvious Cy5 signals from the NPs being observed (Figure 4c and Figure S22). Despite a low amount used to maintain cell viability, the intrinsic green fluorescence from curcumin was also observed inside the cells in the Cur-Tf-PDAAm NP group, confirming their capability to successfully deliver the anti-cancer drug inside the cells. In contrast, only very limited uptake was observed with both the control Cur-POEGA-PDAAm NPs and free curcumin (Figure 4c), further demonstrating the benefit of employing targeted and stimuli-responsive protein–polymer NPs to achieve enhanced drug delivery efficiency for more effective cancer therapeutic applications.

## 3. Conclusions

In conclusion, we have reported the development of reduction-responsive Tf protein–polymer nanoparticles for the targeted and enhanced delivery and release of curcumin to cancer cells for cancer therapy. By employing photo-PISA, stimuli-responsive nanoparticles containing the hydrophilic targeting Tf protein on the shell and curcumin in the hydrophobic core were successfully prepared. When exposed to a reducing environment using 10 mM GSH to mimic the tumour environment, the disulfide linker between the hydrophilic and hydrophobic segments of the Cur-Tf-PDAAm NPs could be cleaved, resulting in the disassembly of the NPs and enhanced drug release. This tumour microenvironment-responsive drug release behaviour of the nanoparticles can potentially reduce off-target toxic effects, thus improving the safety of the Cur-Tf-PDAAm NPs as a promising drug delivery system. The Cur-Tf-PDAAm NPs can enhance cellular uptake and delivery of curcumin to cancer cells through Tf receptor-mediated endocytosis compared to the control NPs (Cur-POEGA-PDAAm) without a targeting protein when tested on MDA-MB-231 breast cancer cells. Consequently, the Cur-Tf-PDAAm NPs were highly effective in inducing cancer cell death compared to the control NPs, demonstrating its potential as a targeted drug delivery system for effective cancer therapy.

## Data Availability

All data are available within the article and its ESI and from the authors upon request.

## Supplementary Materials

The following supporting information can be downloaded at: https://www.mdpi.com/article/10.3390/molecules30040856/s1. Figure S1: Scheme for the synthesis of redox-sensitive SS-CTA; Figure S2: ^1^H NMR (400 MHz, CDCl_3_) of 2-((2-hydroxyethyl)disulfaneyl)ethyl 2-(((butylthio)carbonothioyl)thio)propanoate; Figure S3: ^13^C NMR (100 MHz, CDCl_3_) of 2-((2-hydroxyethyl)disulfaneyl)ethyl 2-(((butylthio)carbonothioyl)thio)propanoate; Figure S4: ^1^H NMR (400 MHz, CDCl_3_) of SS-CTA; Figure S5: ^13^C NMR (100 MHz, CDCl_3_) of SS-CTA; Figure S6: ^1^H NMR (400 MHz, DMSO-d_6_) analysis demonstrating the reduction-responsive cleavage of disulfide bonds of SS-CTA when incubated in 10 mM glutathione; Figure S7: LC-MS analysis demonstrating the redox-responsive cleavage of disulfide bond of SS-CTA when incubated in 10 mM glutathione; Figure S8: ^1^H NMR (400 MHz, CDCl3) of OligoOEGA; Figure S9: SEC trace of OligoOEGA macro-CTA; Figure S10: ^1^H NMR (400 MHz, D2O) of Tf-OligoOEGA; Figure S11: Hydrodynamic size of Tf and Tf-OligoOEGA measured by DLS; Figure S12: MALDI-TOF spectra for Tf and Tf-OligoOEGA; Figure S13: SDS-PAGE of protein markers (1), free Tf (2) and Tf-OligoOEGA (3); Figure S14: ^1^H NMR (400 MHz, DMSO-d6) of Tf-PDAAm NPs; Figure S15: Reaction scheme for the synthesis of Cur-POEGA-PDAAm NPs; Figure S16: ^1^H NMR (CDCl3, 400 MHz) of POEGA; Figure S17: Hydrodynamic size of Cur-POEGA-PDAAm NPs; Figure S18: UV-Vis spectrum of Cur-POEGA-PDAAm NPs (labelled with Cy5) in PBS; Figure S19: DLS analysis of Cur-POEGA-PDAAm NPs incubated with 10 mM glutathione; Figure S20: DLS analysis of Cur-Tf-PDAAm NPs incubated in (a) PBS and (b) PBS containing 10% FBS (without glutathione); Figure S21: (a) RAW264.7 cell viability when incubated with different concentrations of Tf-PDAAm NPs. (b) IC50 determination of Cur-Tf-PDAAm NPs using RAW264.7 cells; Figure S22: Confocal micrographs demonstrating the cellular internalization of Cur-Tf-PDAAm NPs after 1 h incubation with MDA-MB-231 breast cancer cells (scale bar: 10 µm). Refs. [41,42,43] are cited in Supplementary Materials file.
