# Supplementary material for: Self-Assembled Protein–Polymer Nanoparticles via Photoinitiated Polymerization-Induced Self-Assembly for Targeted and Enhanced Drug Delivery in Cancer Therapy
Source: Molecules. 2025 Feb 13;30(4):856. doi: 10.3390/molecules30040856 (PMC11858777; doi:10.3390/molecules30040856)
Supplement: Supplementary file 1 [file molecules-30-00856-s001.zip › molecules-3408892-supplementary.pdf]

Article

Self-Assembled Protein–Polymer Nanoparticles via Photoinitiated Polymerization-Induced Self-Assembly for Targeted and Enhanced Drug Delivery in Cancer Therapy

Gayathri R. Ediriweera [1], Yixin Chang [1], Wenting Yang [1], Andrew K. Whittaker [1,2,*] and Changkui Fu [1,*]

1 Australian Institute for Bioengineering and Nanotechnology, The University of Queensland, St Lucia, QLD 4072, Australia; a.ediriweera@uq.edu.au (G.R.E.); yixin.chang@uq.edu.au (Y.C.); wenting.yang2@student.uq.edu.au (W.Y.)

2 Australian Research Council Centre of Excellence for Green Electrochemical Transformation of Carbon Dioxide, The University of Queensland, St. Lucia, QLD 4072, Australia

* Correspondence: a.whittaker@uq.edu.au (A.K.W.); changkui.fu@uq.edu.au (C.F.)

Experimental Section

Materials

N-(3-dimethylaminopropyl)-N'-ethylcarbodiimide hydrochloride (EDC·HCl), 4-dimethylaminopyridine (97%), 2-hydroxyethyl disulfide, 4-nitrophenyl chloroformate (97%), 2-bromoethylamine hydrobromide (99%), N,N-diisopropylethylamine (>99%), methacryloyl chloride (97%), sodium azide (>99.5%), azobisisobutyronitrile (AIBN), Eosin Y (99%), N,N,N',N'',N'''-pentamethyldiethylenetriamine (PMDETA, 99%), transferrin human (>98%), poly(ethylene glycol) methyl ether acrylate (M_n = 480 g/mol) were purchased from Sigma-Aldrich and used as received. 2-(((Butylthio)carbonothioyl)thio)propanoic acid (BTPA) and N-(2-azidoethyl)methacrylamide were synthesized according to the previous reports.[1-3]

Nuclear Magnetic Resonance (NMR) ^{1}H and ^{13}C NMR spectra were recorded on a Bruker Avance 400 MHz spectrometer at 298 K using $CDCl_3$, DMSO-d_6 or D_2O as solvents. All chemical shifts are reported in ppm (δ) relative to tetramethylsilane (TMS).

Size Exclusion Chromatography (SEC) The molecular weight (M_n) and the molecular weight distribution (molar mass dispersity, $Đ_M = M_w/M_n$) of the oligomer were determined by SEC using a Waters Alliance 2690 Separation Module equipped with a Watter 2414 differential refractive index (RI) detector, Waters 2489 UV/Visible detector, Waters 717 Plus Autosampler and Waters 1515 Isocratic HPLC pump. THF was used as the mobile phase with a flow rate of 1 mL/min. The system was calibrated using polystyrene standards with molecular weights ranging from 6.82×10^2 g/mol to 1.67×10^6 g/mol. The oligomer was dissolved in THF, filtered through a PTFE membrane (0.45 μm pore size), and then subjected to injection.

Tf and Tf-OligoOEGA were characterized on an Agilent Technologies 1260 Infinity Aqueous GPC system equipped with Agilent software to operate the system. The instrument was connected to RI, UV, viscosity and MALS detectors. Two PL aqueous-OH mixed, 8 micron columns were used to analyse the samples. The columns were kept at 40 °C with the flow rate of 1 mL/min during the analysis. PEO molecular weight standards were used to create a calibration curve to achieve the molecular weights of the samples.

Matrix-assisted Laser Desorption/ionization-time of Flight (MALDI-TOF) MALDI-TOF was performed on a Bruker Autoflex Speed mass spectrometer. Samples were dissolved in mixed solvent of ethanol, acetone, and trifluoroacetic acid (6:3:0.1, v/v/v) with a concentration of 0.5 mg/mL. A 1:1 mix of α-cyano-4-hydroxycinnamic acid (CHCA) and 2,5-dihydroxy benzoic acid (DHBA) was used as the matrix for all the measurements.

Dynamic Light Scattering (DLS) DLS measurements were conducted using a Malvern Instrument Zetasizer nano series instrument equipped with a 4.0 mW He–Ne laser operating at 633 nm and a detection angle of 173°. The number-weighted hydrodynamic diameter was obtained from analysis of the autocorrelation functions using the method of cumulants. At least three measurements at 25 °C or 37 °C for nanoparticle stability test were made for each sample with an equilibrium time of 2 min before starting the measurement.

UV-Vis Spectroscopy The UV-Vis spectra were recorded on a Varian Cary 4000 UV-vis spectrophotometer. Absorbance maxima were recorded at 309 nm, 425 nm and 647 nm for the RAFT CTA, curcumin and Cy5 absorbance respectively.

Mass spectrometry Mass spectrometry analysis was carried out using Waters Micromass Quattro mass spectrometer in positive mode using direct injection method. All the samples were dissolved in methanol at a concentration of 0.1 mg/mL and filtered, and methanol or acetonitrile was used as the mobile phase throughout the run.

SDS-PAGE All SDS-PAGE runs were carried out using 4-20% precast polyacrylamide gels in Tris/Glycine/SDS running buffer at 100 V.

Cell culture All products that are related to cell biology including Dulbecco's modified eagle medium (DMEM), fetal bovine serum (FBS), penicillin-streptomycin antibiotic solution, trypsin, trypan blue solution, CellTiter 96®Aqueous one solution cell proliferation assay (MTS) and phosphate-buffered saline (PBS) were purchased from Sigma-Aldrich. Live breast cancer cells (ATCC designation MDA-MB-231) were maintained in DMEM media, that were supplemented with 10% (v/v) FBS, 100 U/mL penicillin, 100 μg/mL Streptomycin and 2 mM L-glutamine and incubated at 37 °C in a humidified atmosphere of 5% CO_2 in air.

Confocal microscopy Confocal microscopy for cellular internalization was performed using Zeiss LSM 710 confocal laser scanning microscope.

Synthesis of redox-sensitive CTA (SS-CTA)

BTPA (2.5 g, 10.5 mmol) and EDC.HCl (3.0 g, 15.7 mmol) were dissolved in CH_2Cl_2 (30 mL) in an ice bath. 2-hydroxyethyl disulfide (1.8 g, 11.5 mmol) and 4-dimethylaminopyridine (0.2 g, 2.1 mmol) were then added into the above solution in 10 mL of CH_2Cl_2 and the reaction was allowed to occur overnight at room temperature. Then, the organic phase was washed with water (50 mL × 2), dried over anhydrous magnesium sulfate, filtered and the solvent was removed under vacuum to obtain the crude product. The crude product was then purified using silica column chromatography using hexane: ethyl acetate (8:2 followed by 7:3) as the mobile phase to obtain the pure product (2.1 g, 54%). ^1H NMR ($CDCl_3$, 400 MHz) 4.79 (q, CH), 4.40 (t, CH_2), 3.86 (t, CH_2), 3.34 (t, CH_2), 2.91 (t, CH_2), 2.86 (t, CH_2), 1.66 (m, CH_2), 1.58 (d, CH_3), 1.41 (m, CH_2), 0.92 (t, CH_3) ^{13}C NMR ($CDCl_3$, 100 MHz) 221.96 (C=S), 171.02 (C=O), 63.51 (O-CH_2), 60.37 (O-CH_2), 47.87 (CH), 41.52 (S-CH_2), 37.17 (S-CH_2), 36.77 (S-CH_2), 29.88 (CH_2), 22.13 (CH_2), 16.78 (CH_3), 13.64 (CH_3).

2-((2-hydroxyethyl)disulfaneyl)ethyl 2-(((butylthio)carbonothioyl)thio)propanoate (1.1 g, 2.9 mmol) and triethylamine (0.6 g, 5.8 mmol) were dissolved in CH_2Cl_2 (20 mL) in an ice bath. 4-Nitrophenyl chloroformate (0.8 g, 3.8 mmol) was then added into the above solution in 5 mL of CH_2Cl_2 and the reaction was allowed to occur overnight at room temperature. Then, the residue was filtered, and the solvent was concentrated under vacuum to obtain the crude product. The crude product was then purified using silica column chromatography using hexane: ethyl acetate (6:4) as the mobile phase to obtain the pure product (0.8 g, 47%). ^1H NMR ($CDCl_3$, 400 MHz) 8.28 (d, CH in Ph), 7.39 (d, CH in Ph), 4.81 (q, CH), 4.54 (t, CH_2), 4.41 (t, CH_2), 3.35 (t, CH_2), 3.06 (t, CH_2), 2.96 (t, CH_2), 1.67 (m, CH_2), 1.60 (d, CH_3), 1.42 (m, CH_2), 0.92 (t, CH_3) ^{13}C NMR ($CDCl_3$, 100 MHz) 222.01 (C=S), 171.06 (C=O), 155.41 (C in Ph), 152.27 (C=O), 145.51 (C in Ph), 125.32 (CH in Ph), 121.77 (CH in Ph), 66.94 (O-CH_2), 63.33 (O-CH_2), 47.88 (CH), 42.33 (S-CH_2), 40.59 (S-CH_2), 37.18 (S-CH_2), 30.09 (CH_2), 22.13 (CH_2), 16.78 (CH_3), 13.64 (CH_3).

Synthesis of OligoOEGA

SS-CTA (0.3 g, 0.5 mmol), poly(ethylene glycol) methyl ether acrylate (0.7 g, 1.5 mmol) and AIBN (0.02 g, 0.1 mmol) were dissolved in 2 mL of 1,4-dioxane in a 10 mL tube. The tube was sealed and degassed with argon for 30 min. The reaction mixture was then put in an oil bath preheated at 70 °C and the stirring was continued for 6 h. Subsequently, the polymerization was quenched by exposing to air and the monomer conversion was determined through ^1H NMR. After removing the solvent, the oligomer was purified by precipitating in diethyl ether (50 mL) three times using CH_2Cl_2 to dissolve the polymer. The final product was dried under vacuum to obtain a yellow sticky oil.

Synthesis of Tf-OligoOEGA

For the synthesis of Tf-OligoOEGA, transferrin protein (0.1 g, 0.0013 mmol) was dissolved in 10 mL of PBS buffer (pH = 7.4) in a round bottom flask. The OligoOEGA (0.04 g, 0.013 mmol) dissolved in 5 mL of PBS buffer was added to the protein solution dropwise. The reaction was allowed to occur under cold conditions for 48 h. The final product was obtained by dialysis in water (10 kDa MWCO) followed by lyophilisation as an off-white powder.

Synthesis of Tf-PDAAm NPs

For the synthesis of NPs, Tf-OligoOEGA (0.02 g, 0.0002 mmol), diacetone acrylamide (0.02 g, 0.14 mmol), eosin Y (0.00003 g, 0.00005 mmol) and PMDETA (0.00004 g, 0.0002 mmol) were mixed with 0.7 mL of PBS buffer (pH = 7) in a glass vial. The vial was sealed and was degassed with argon for 30 min. The solution was irradiated under blue LED light (λ_{max} = 470 nm) while being gently stirred. After 5 h, the polymerization (~80% conversion) was stopped by exposing the solution to air and by turning off the LED light. The product was purified by dialysis in PBS to yield the Tf-PDAAm NPs in suspension.

Synthesis of Cur-Tf-PDAAm NPs

For the synthesis of drug loaded NPs, Tf-OligoOEGA (0.02 g, 0.0002 mmol), diacetone acrylamide (0.02 g, 0.14 mmol), curcumin (0.002 g, 0.005 mmol) eosin Y (0.00003 g, 0.00005 mmol) and PMDETA (0.00004 g, 0.0002 mmol) were mixed with 0.7 mL of PBS buffer (pH = 7) in a glass vial. The vial was sealed and was degassed with argon for 30 min. The solution was irradiated under blue LED light (λmax = 470 nm) while being gently stirred. After 5 h, the polymerization was stopped by exposing the solution to air and by turning off the LED light. The product was purified by dialysis in PBS to yield the Cur-Tf-PDAAm NPs in suspension. The drug loading efficiency was calculated by dissolving 10 µL of NP suspension in DMSO to release the curcumin followed by measuring the UV-Vis absorbance at 425 nm and comparing it against a calibration curve derived from free curcumin in DMSO.

Drug loading efficiency (%) = (Encapsulated curcumin)/(Total curcumin) × 100%

Synthesis of POEGA

Poly(ethylene glycol) methyl ether acrylate (2.0 g, 4.1 mmol), SS-CTA (0.01 g, 0.02 mmol), N-(2-azidoethyl)methacrylamide (0.02 g, 0.1 mmol) and AIBN (0.001 g, 0.007 mmol) were dissolved in 4 mL of DMF in a 20 mL tube. The tube was sealed and degassed with argon for 30 min. The reaction mixture was then put in an oil bath preheated at 70 °C and the stirring was continued for 24 h. Subsequently, the polymerization was quenched by exposing to air and the monomer conversion was

determined through ^{1}H NMR (~99% conversion). After removing the solvent, the polymer was purified by precipitating in diethyl ether (50 mL) three times to obtain the final product as a yellow oil.

Synthesis of Cur-POEGA-PDAAm NPs

For the synthesis of drug loaded NPs, POEGA (0.099 g, 0.001 mmol), diacetone acrylamide (0.04 g, 0.25 mmol), curcumin (0.007 g, 0.02 mmol) eosin Y (0.0001 g, 0.0002 mmol) and PMDETA (0.0002 g, 0.001 mmol) were mixed with 2 mL of PBS buffer (pH = 7) in a glass vial. The vial was sealed and was degassed with argon for 30 min. The solution was irradiated under blue LED light (λ_{max} = 470 nm) while being gently stirred. After 5 h, the polymerization was stopped by exposing the solution to air and by turning off the LED light. The product was purified by dialysis in PBS to yield the Cur-POEGA-PDAAm NPs in suspension. The drug loading efficiency was calculated by dissolving 10 μL of NP suspension in DMSO to release the curcumin followed by measuring the UV-Vis absorbance at 425 nm and comparing it against a calibration curve derived from free curcumin in DMSO.

Fluorescent dye labelling of NPs

Cur-Tf-PDAAm NPs (500 μL from the suspension) was reacted with 0.2 mg of Cy5 NHS ester dissolved in 10 μL of DMSO under cold conditions for 24 h. The reaction mixture was then transferred to a 10 kDa dialysis bag and purified by dialysis in PBS under cold conditions.

The same procedure was repeated with Cur-POEGA-PDAAm NPs with sulfo-Cy5-DBCO under cold conditions to fluorescently label the control NPs.

Disulfide bond cleavage kinetics of the SS-CTA

SS-CTA (4.3 mg, 0.008 mmol) and glutathione (2.5 mg, 0.008 mmol) were dissolved in 0.8 mL of DMSO-d_6 (final glutathione concentration: 10 mM). The sample was subjected to ^{1}H NMR at different time points in order to determine the cleavage kinetics.

Drug release and stability of NPs in a reducing environment

Cur-Tf-PDAAm and Cur-POEGA-PDAAm NPs were diluted in a glutathione containing PBS solution in 1:4 NP suspension to glutathione solution ratio (final glutathione concentration: 10 mM) and the change in particle size was measured through DLS at different time points. In order to determine the drug release (%), aliquots from NP suspension in glutathione were taken out at different time points, centrifuged at 13.4 rcf for 20 min to obtain free curcumin, resuspended in DMSO and was quantified by measuring the absorbance at 425 nm using plate reader and compared it against the total amount of drug in the aliquoted volume derived using a calibration curve for curcumin in DMSO.

In vitro cytotoxicity of NPs

The *in vitro* cytotoxicity of NPs was investigated with MDA-MB-231 cells using MTS assay. First, the cells were seeded in 96-well plates with the same density (10^4 cells per well) and were incubated for 24 h at 37 °C. Cur-Tf-PDAAm and Cur-POEGA-PDAAm, and Tf-PDAAm containing varying amounts of curcumin (6.25, 12.5, 25, 50, 100 and 200 μM curcumin) were then added in serum-supplemented tissue culture media and again incubated for 48 h at 37 °C. Tf-PDAAm NPs (bare Tf NPs without curcumin) and free curcumin dissolved in 2.5% DMSO containing media were also incubated with cells for comparison. After 48 h, the cells were washed twice with serum-supplemented tissue culture media followed by the incubation with 100 μL of MTS solution (20 μL CellTiter 96 Aqueous One Solution Reagent and 80 μL of tissue culture medium) for 2 h, and the absorbance was measured at 490 nm using a microplate reader.

Cell viability = (Sample absorbance-Blank absorbance)/(Cell absorbance-Blank absorbance) × 100

In vitro cellular association and receptor blocking study

For cellular association and uptake studies, MDA-MB-231 cells were added into autoclaved Eppendorf tubes at a density of 10^5 cells per tube. Afterwards, Cur-Tf-PDAAm and Cur-POEGA-PDAAm NPs containing 2 μg of curcumin and 2 μg free curcumin were added into separate cell tubes and were incubated at 4 ˚C. The same experiment was performed at 37 ˚C as well. After incubating for 1 h, the tubes were centrifuged and washed 3 times with PBS containing 2% FBS. The cells were then resuspended in 150 μL of FACS wash (PBS with 2-3% FBS) for flow cytometry analysis. The cell samples were subsequently analysed using fluorescence-activated cell sorting (FACS) on a Cytoflex S (Beckman Coulter). Data was acquired for 10,000 events measuring Cy5 fluorescence intensity (APC) along with the forward and side scatter. Untreated cells were used to gate the population of viable cells.

For blocking experiment, MDA-MB-231 cells were added into autoclaved Eppendorf tubes at a density of 10^5 cells per tube. Afterwards, the cells were incubated with an excess amount of free Tf protein (0.8 mg of Tf per tube) and Cur-Tf-PDAAm and Cur-POEGA-PDAAm NPs containing 2 μg of curcumin at 4 ˚C. After incubating for 1 h, the tubes were centrifuged and washed 3 times with PBS containing 2% FBS. The cells were then resuspended in 150 μL of FACS wash (PBS with 2-3% FBS) for flow cytometry analysis. The cell samples were subsequently analysed using fluorescence-activated cell sorting (FACS) on a Cytoflex S (Beckman Coulter). Data was acquired for 10,000 events measuring Cy5 fluorescence intensity (APC) along with the forward and side scatter. Untreated cells were used to gate the population of viable cells.

In vitro cellular uptake and internalization

MDA-MB-231 cells were seeded in a MatTek glass bottom dish (35 mm Dish, No. 1.5 Coverslip, 14 mm Glass Diameter) at a density of 10^5 cells per dish and were incubated overnight. The media was removed and replaced with media containing Cur-Tf-PDAAm and Cur-POEGA-PDAAm NPs

containing 15 μM curcumin. The cells were then incubated with NPs for 5 h and were washed three times with media. In order to stain the nuclei, the cells were again incubated for 10 min with media containing Hoechst (as per supplier protocol). The cells were washed three times with PBS containing 5% FBS and were imaged using Zeiss 710 confocal microscope at 63× oil objective.

Supporting data

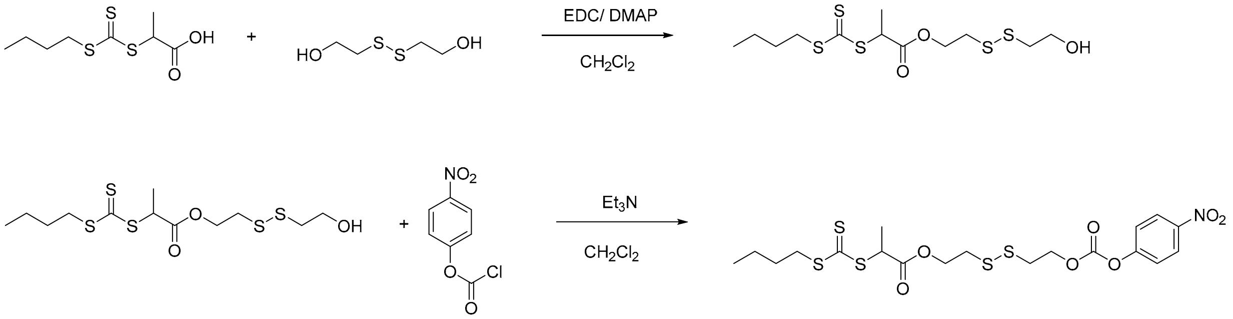

Figure S1. Scheme for the synthesis of redox-sensitive SS-CTA.

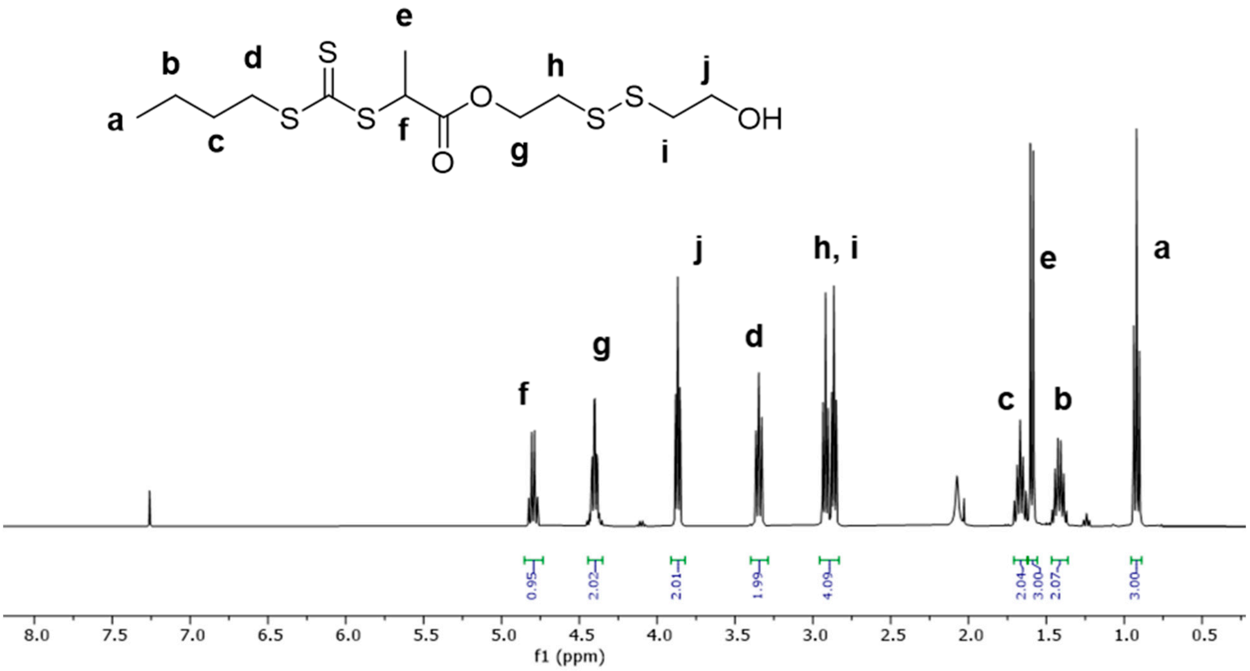

Figure S2. ^{1}H NMR (400 MHz, $CDCl_3$) of 2-((2-hydroxyethyl)disulfaneyl)ethyl 2-(((butylthio)carbonothioyl)thio)propanoate.

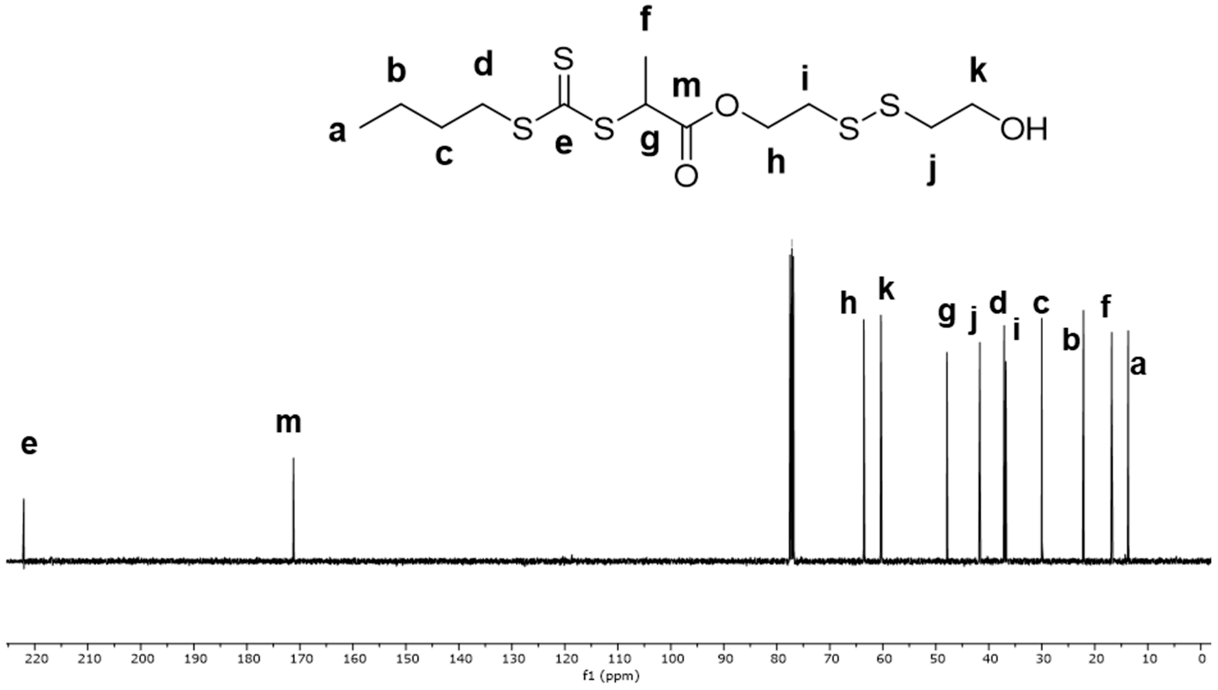

Figure S3. ^{13}C NMR (100 MHz, $CDCl_3$) of 2-((2-hydroxyethyl)disulfaneyl)ethyl 2-(((butylthio)carbonothioyl)thio)propanoate.

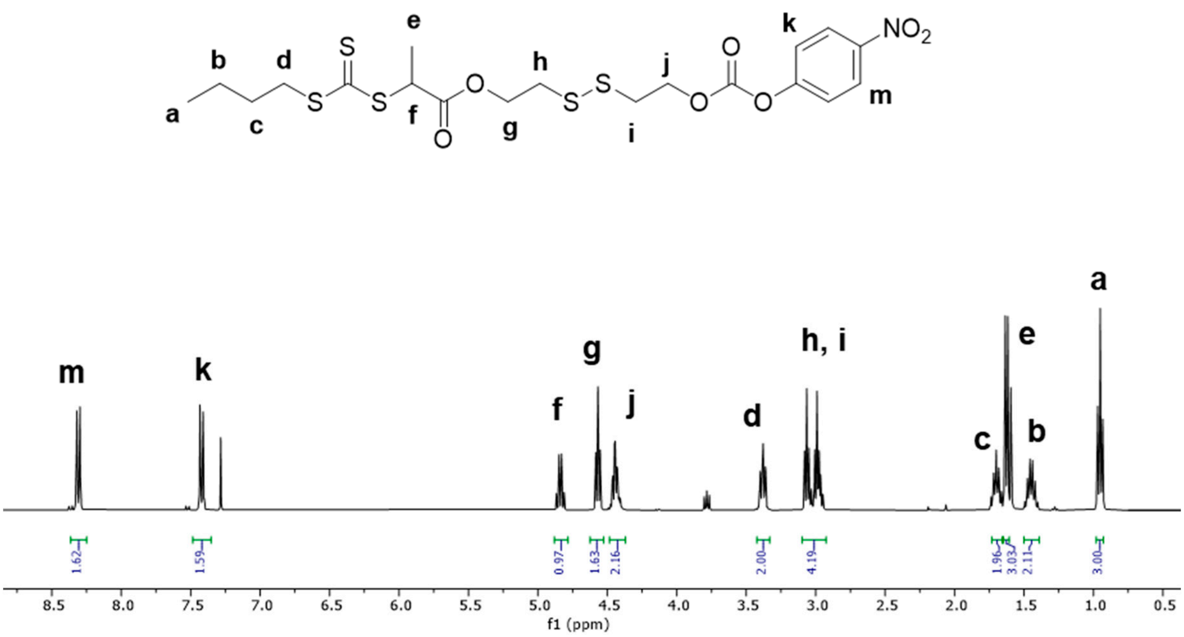

Figure S4. ^{1}H NMR (400 MHz, $CDCl_3$) of SS-CTA.

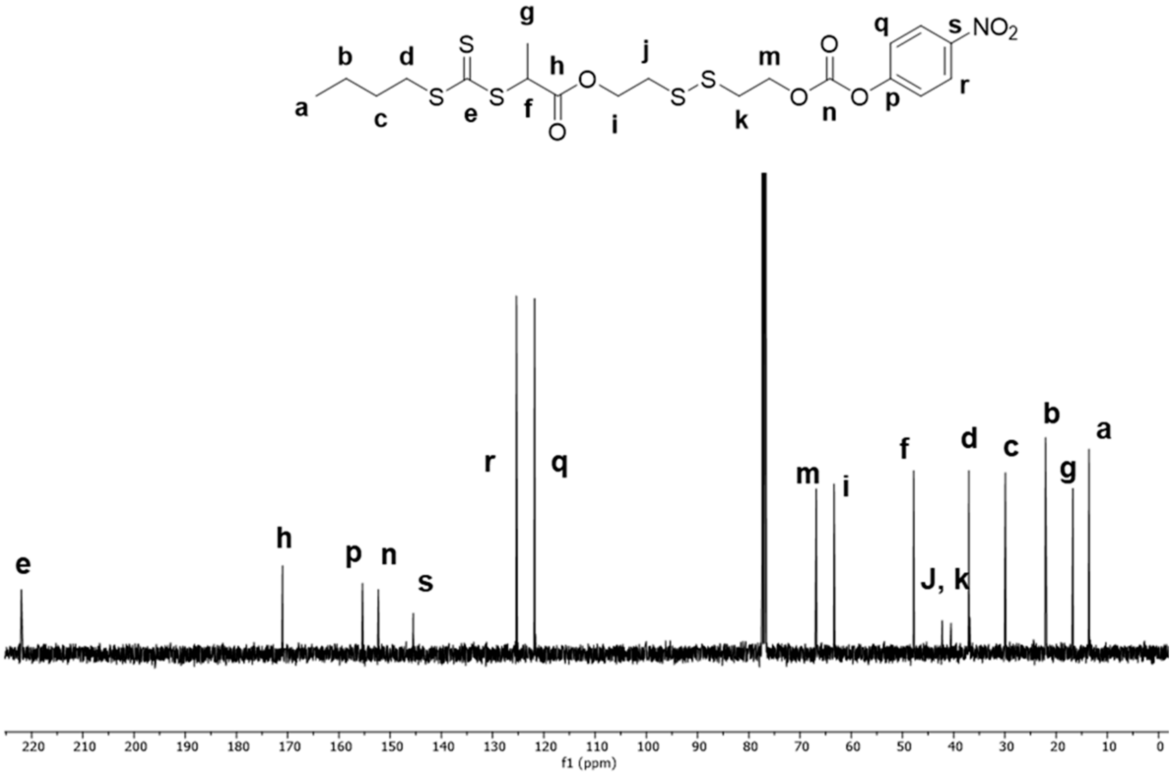

Figure S5. ^{13}C NMR (100 MHz, $CDCl_3$) of SS-CTA.

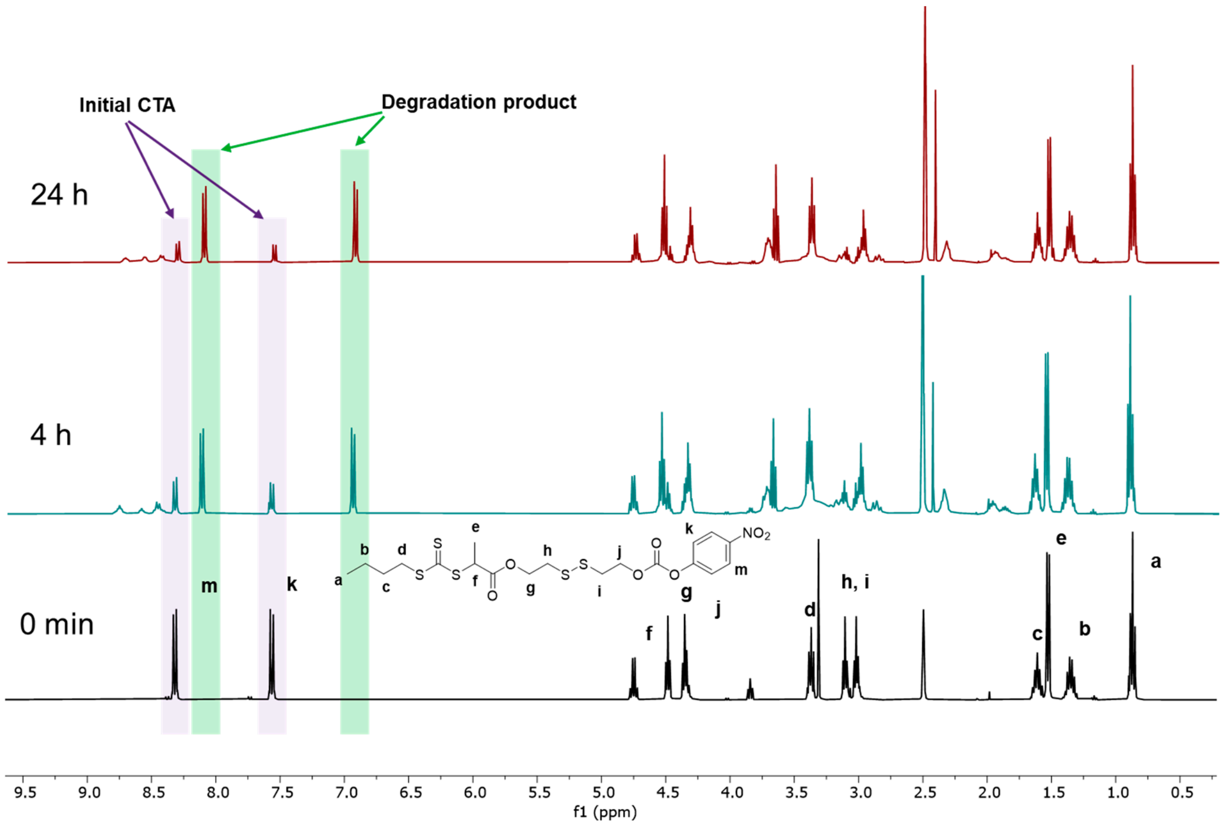

Figure S6. ^{1}H NMR (400 MHz, DMSO-d_6) analysis demonstrating the reduction-responsive cleavage of disulfide bonds of SS-CTA when incubated in 10 mM glutathione.

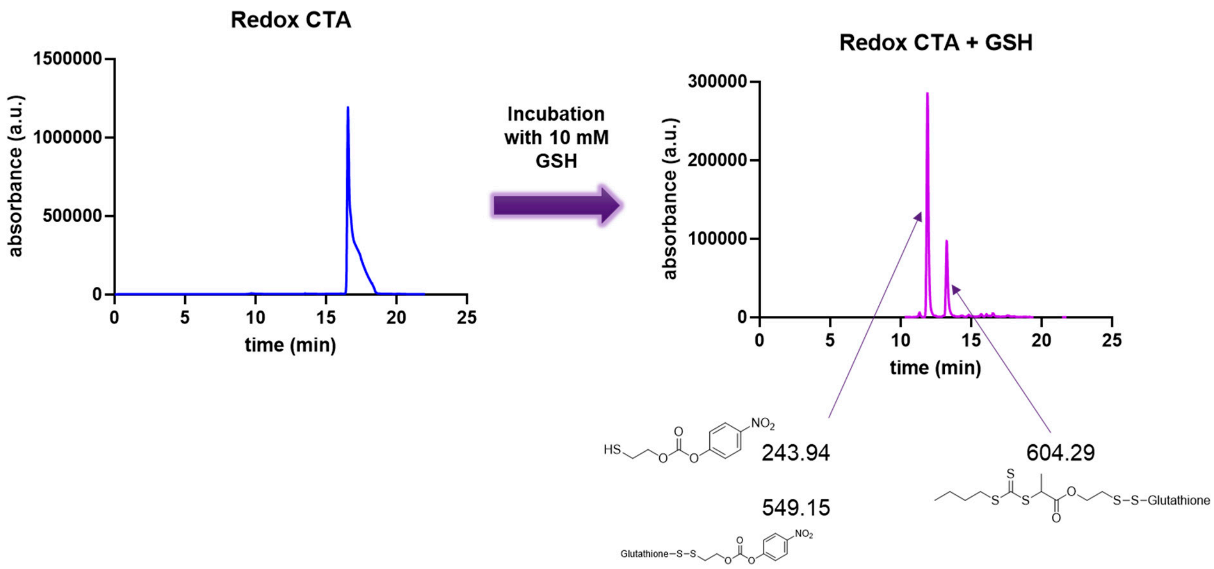

Figure S7. LC-MS analysis demonstrating the redox-responsive cleavage of disulfide bond of SS-CTA when incubated in 10 mM glutathione.

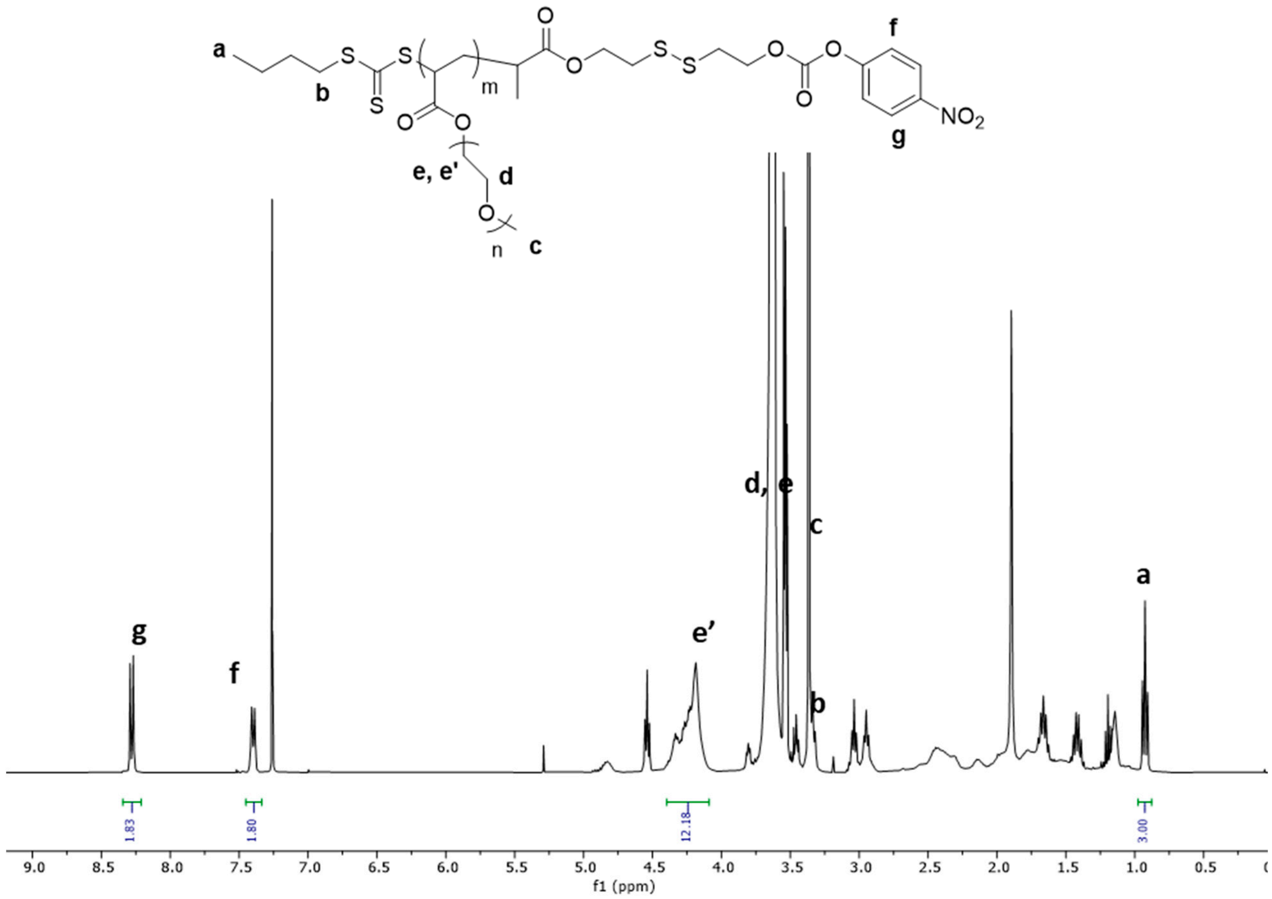

Figure S8. ^{1}H NMR (400 MHz, $CDCl_3$) of OligoOEGA.

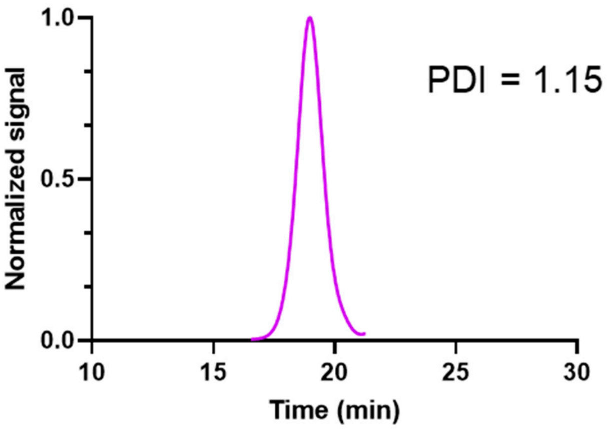

Figure S9. SEC trace of OligoOEGA macro-CTA.

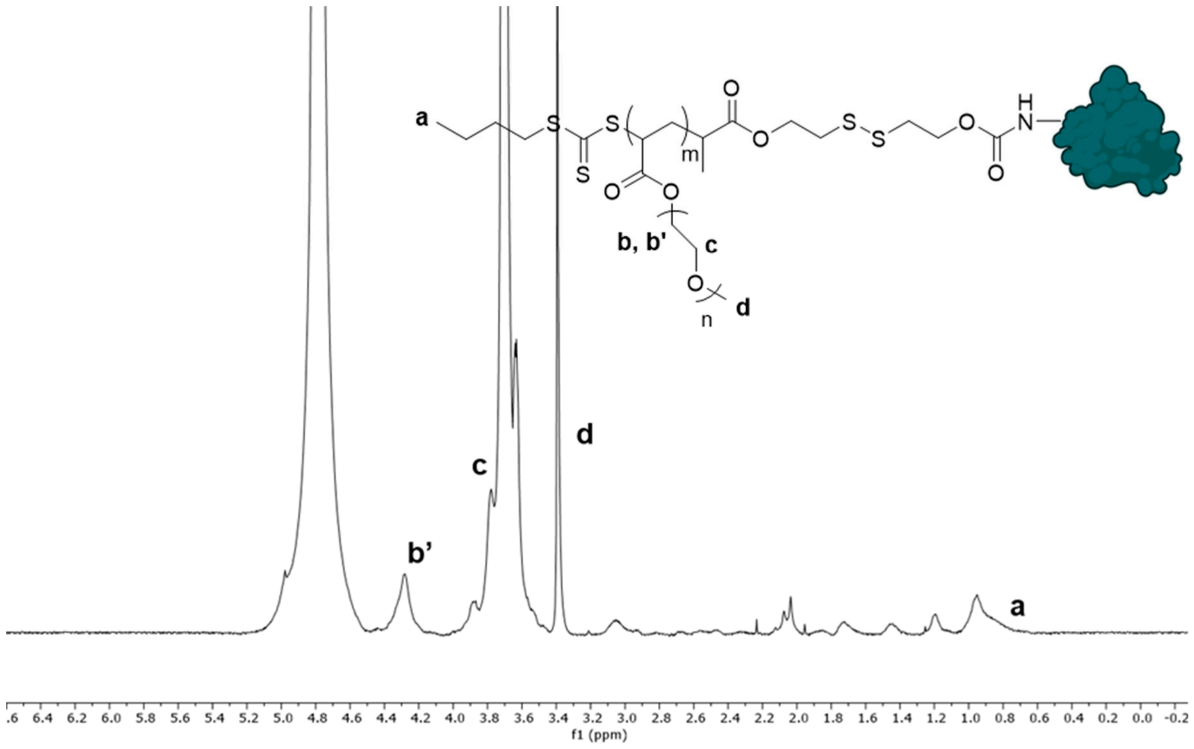

Figure S10. ^{1}H NMR (400 MHz, D_2O) of Tf-OligoOEGA.

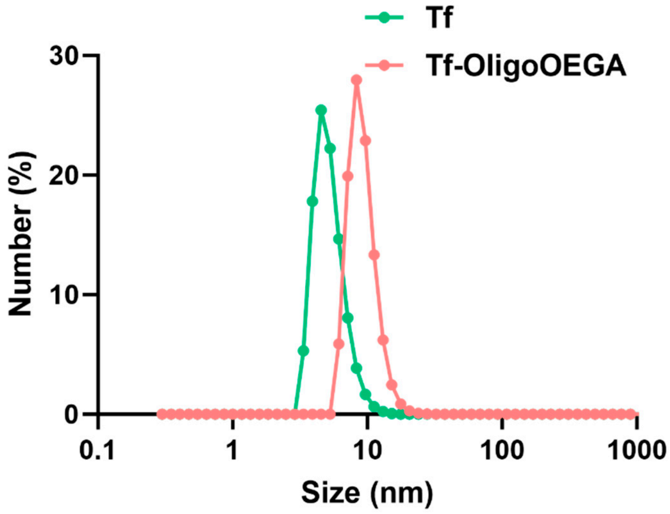

Figure S11. Hydrodynamic size of Tf and Tf-OligoOEGA measured by DLS.

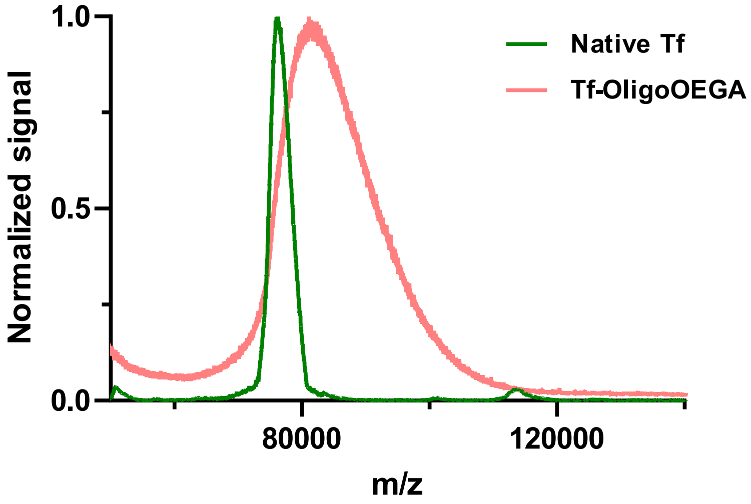

Figure S12. MALDI-TOF spectra for Tf and Tf-OligoOEGA.

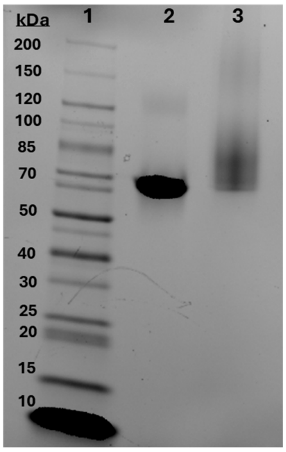

Figure S13. SDS-PAGE of protein markers (1), free Tf (2) and Tf-OligoOEGA (3).

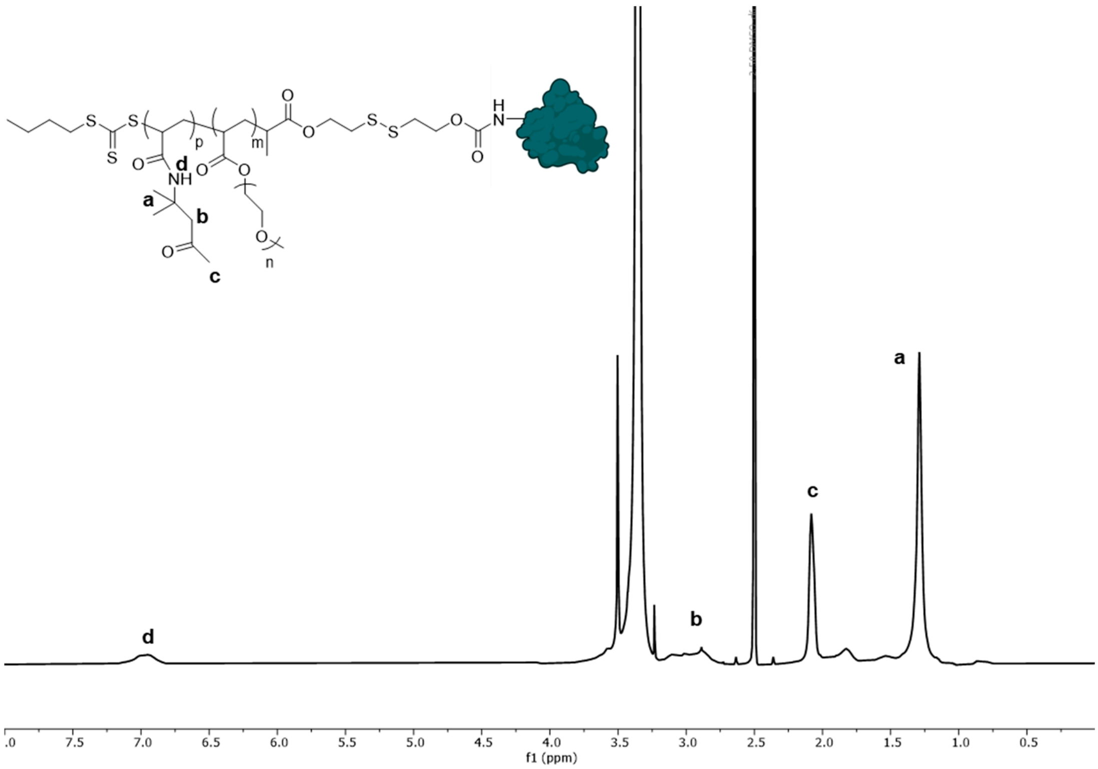

Figure S14: ^{1}H NMR (400 MHz, DMSO-d$_6$) of Tf-PDAAm NPs.

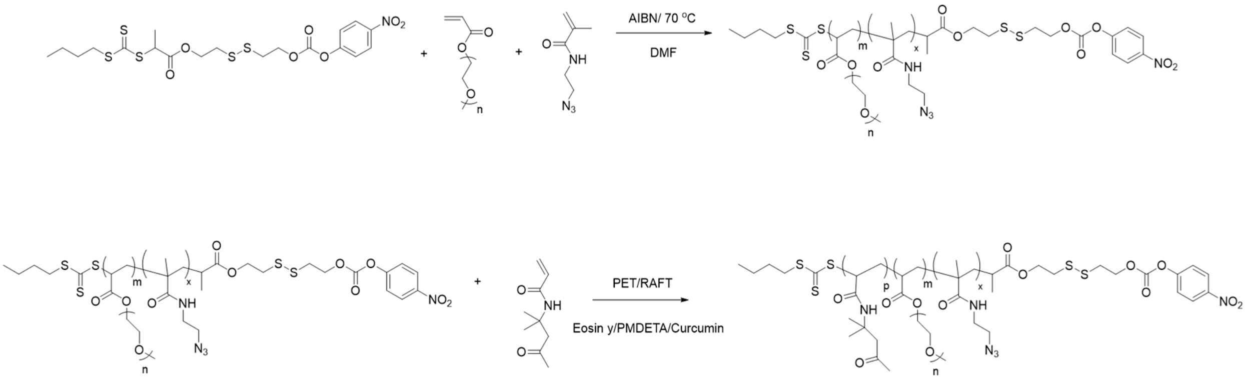

Figure S15. Reaction scheme for the synthesis of Cur-POEGA-PDAAm NPs.

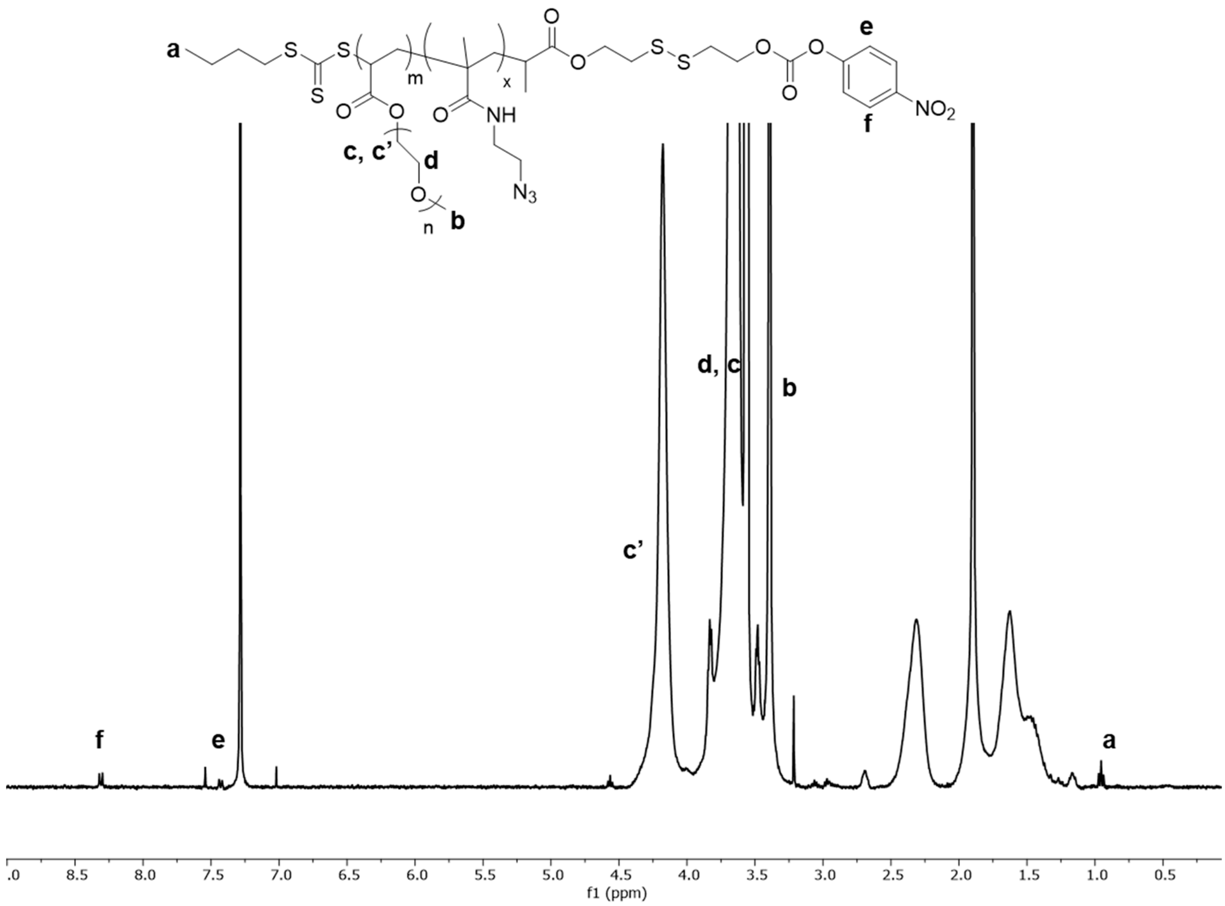

Figure S16. ^1H NMR ($CDCl_3$, 400 MHz) of POEGA.

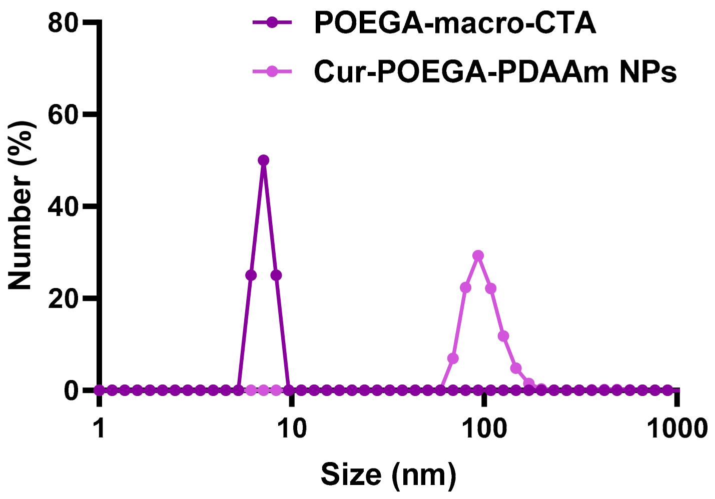

Figure S17. Hydrodynamic size of Cur-POEGA-PDAAm NPs.

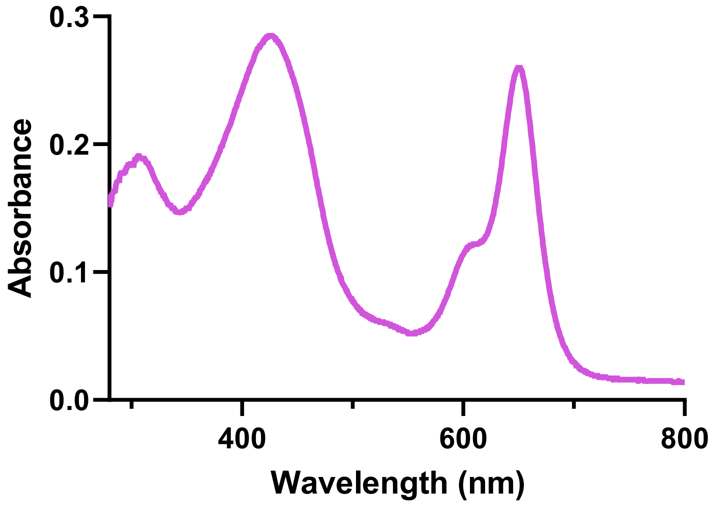

Figure S18. UV-Vis spectrum of Cur-POEGA-PDAAm NPs (labelled with Cy5) in PBS.

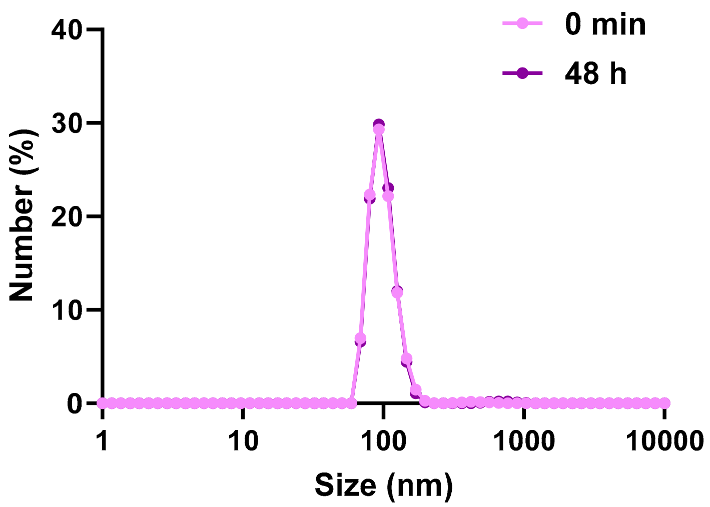

Figure S19. DLS analysis of Cur-POEGA-PDAAm NPs incubated with 10 mM glutathione.

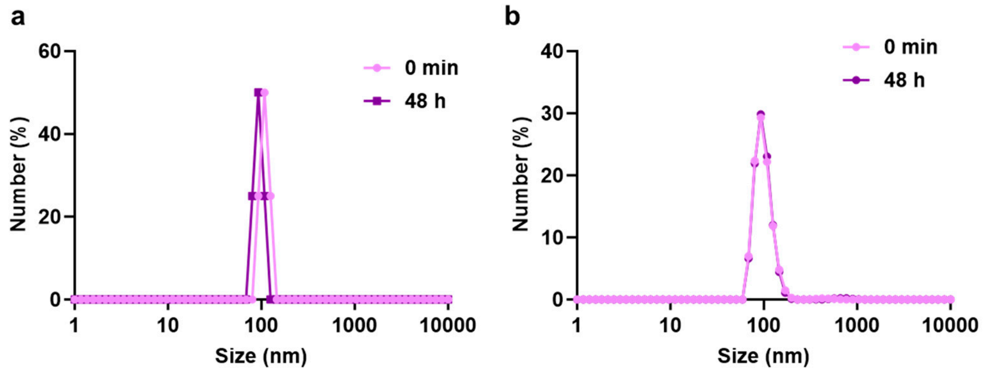

Figure S20. DLS analysis of Cur-Tf-PDAAm NPs incubated in a) PBS and b) PBS containing 10% FBS (without glutathione).

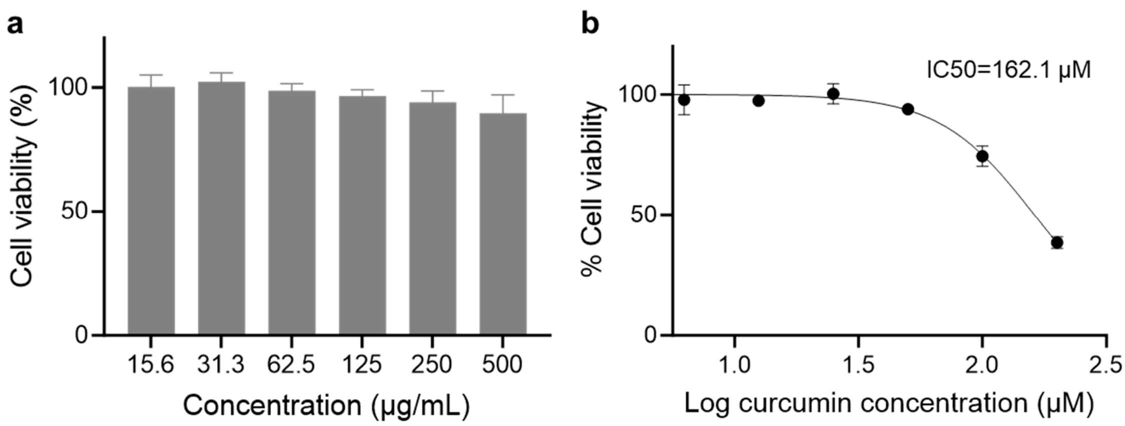

Figure S21. (a) RAW264.7 cell viability when incubated with different concentrations of Tf-PDAAm NPs. (b) IC_{50} determination of Cur-Tf-PDAAm NPs using RAW264.7 cells.

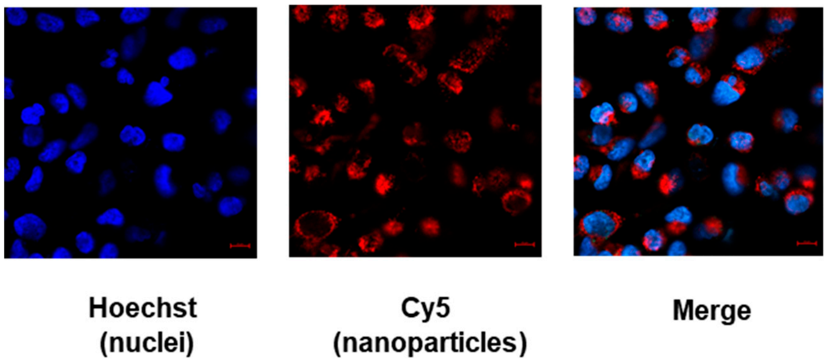

Figure S21. Confocal micrographs demonstrating the cellular internalization of Cur-Tf-PDAAm NPs after 1 h incubation with MDA-MB-231 breast cancer cells (scale bar: 10 μm).

References

1. R. Wang, C. L. McCormick and A. B. Lowe, *Macromolecules*, 2005, **38**, 9518-9525.
2. C. Fu, J. Xu, M. Kokotovic and C. Boyer, *ACS Macro Letters*, 2016, **5**, 444-449.
3. G. R. Ediriweera, Y. Chang, Q. Wang, Y. Gong, D. T. Akhter, H. Pang, F. Y. Han, C. Chen, A. K. Whittaker and C. Fu, *Chemistry of Materials*, 2023, **35**, 7252-7265.
